# *IL2RA* Genetic Heterogeneity in Multiple Sclerosis and Type 1 Diabetes Susceptibility and Soluble Interleukin-2 Receptor Production

**DOI:** 10.1371/journal.pgen.1000322

**Published:** 2009-01-02

**Authors:** Lisa M. Maier, Christopher E. Lowe, Jason Cooper, Kate Downes, David E. Anderson, Christopher Severson, Pamela M. Clark, Brian Healy, Neil Walker, Cristin Aubin, Jorge R. Oksenberg, Stephen L. Hauser, Alistair Compston, Stephen Sawcer, The International Multiple Sclerosis Genetics Consortium, Philip L. De Jager, Linda S. Wicker, John A. Todd, David A. Hafler

**Affiliations:** 1 Division of Molecular Immunology, Center for Neurologic Diseases, Brigham and Women's Hospital and Harvard Medical School, Boston, Massachusetts, United States of America; 2 Program in Medical and Population Genetics, Broad Institute, Massachusetts Institute of Technology and Harvard University, Cambridge, Massachusetts, United States of America; 3 Juvenile Diabetes Research Foundation/Wellcome Trust Diabetes and Inflammation Laboratory, Cambridge Institute for Medical Research, University of Cambridge, Cambridge, United Kingdom; 4 Biostatistics Center, Massachusetts General Hospital, Boston, Massachusetts, United States of America; 5 Department of Neurology, Brigham and Women's Hospital, Boston, Massachusetts, United States of America; 6 University of California San Francisco, San Francisco, California, United States of America; 7 Department of Clinical Neurosciences, Addenbrooke's Hospital, University of Cambridge School of Clinical Medicine, Cambridge, United Kingdom; 8 Harvard Medical School/Partners Healthcare Center for Genetics and Genomics, Boston, Massachusetts, United States of America; The University of Queensland, Australia

## Abstract

Multiple sclerosis (MS) and type 1 diabetes (T1D) are organ-specific autoimmune disorders with significant heritability, part of which is conferred by shared alleles. For decades, the Human Leukocyte Antigen (HLA) complex was the only known susceptibility locus for both T1D and MS, but loci outside the HLA complex harboring risk alleles have been discovered and fully replicated. A genome-wide association scan for MS risk genes and candidate gene association studies have previously described the *IL2RA* gene region as a shared autoimmune locus. In order to investigate whether autoimmunity risk at *IL2RA* was due to distinct or shared alleles, we performed a genetic association study of three *IL2RA* variants in a DNA collection of up to 9,407 healthy controls, 2,420 MS, and 6,425 T1D subjects as well as 1,303 MS parent/child trios. Here, we report “allelic heterogeneity” at the *IL2RA* region between MS and T1D. We observe an allele associated with susceptibility to one disease and risk to the other, an allele that confers susceptibility to both diseases, and an allele that may only confer susceptibility to T1D. In addition, we tested the levels of soluble interleukin-2 receptor (sIL-2RA) in the serum from up to 69 healthy control subjects, 285 MS, and 1,317 T1D subjects. We demonstrate that multiple variants independently correlate with sIL-2RA levels.

## Author Summary

Multiple sclerosis (MS) and type 1 diabetes (T1D) are common, organ-specific inflammatory disorders that continue to increase in global prevalence. The processes leading to both T1D and MS are genetically determined and are thought to involve an autoimmune mechanism. After decades of research into the genetic basis of both MS and T1D, the Human Leukocyte Antigen Complex was the only known susceptibility locus for both T1D and MS. The sequencing of the human genome followed by the generation of the haplotype map, a catalogue of common genetic variation, has allowed the elucidation of allelic variants that define disease risk. Our groups have performed genome-wide association scans and candidate gene studies in both T1D and MS; the final results have identified loci outside the HLA harboring fully replicated risk alleles. Here, we show that the IL-2RA gene encoding a critical regulator of immune responses, the alpha chain of the interleukin-2 receptor, harbors variants that differentially confer risk to MS and T1D. In addition, several independent variants correlate with levels of soluble interleukin-2 receptor in the serum. This finding has critical implications for the field of complex disease genetics as it emphasizes the caution that must be taken when interpreting results for such a complex region with multiple susceptibility alleles.

## Introduction

Recent genome wide association (GWA) and candidate gene studies across human autoimmune disease revealed a shared genetic architecture [1]. These include *PTPN22*, associated with systemic lupus erythematosus (SLE), rheumatoid arthritis (RA), T1D, and Graves' Disease (GD) [2], *STAT4*, associated with SLE and RA [3], and the *IL7R* and *KIAA0350* gene regions, which are shared between T1D and MS [4]–[6]. The *IL2RA* gene region is shared among T1D [7]–[9], MS [6],[10], GD [11], SLE [12] and RA [13],[14]. This overlap of risk loci among autoimmune diseases raises the possibilities that either (1) the same alleles, (2) non-shared, disease-specific alleles, or perhaps (3) a combination of shared and disease-specific alleles confer risk to each of the individual diseases.

In the IL-2RA gene region, a GWA study for MS risk alleles and a large-scale fine-mapping study in T1D provided compelling evidence for a shared autoimmunity locus. A GWA study for MS susceptibility genes performed by The International Multiple Sclerosis Genetics Consortium [6] highlighted two SNPs in the IL-2RA gene: rs12722489 (Odds Ratio (OR) for minor allele  = 0.80; 95% confidence interval (c.i.) = 0.74–0.86, *P* = 2.96×10^−8^) and rs2104286 (OR = 0.84; 95% c.i. = 0.79–0.90; *P* = 2.16×10^−7^). These are in moderate linkage disequilibrium (LD) with each other (*r* ^2^ = 0.62; [6]). The MS association at *IL2RA* has recently been replicated in over 600 multiplex families from Canada (rs12722489, *P* = 0.009; OR = 0.81; 95% c.i. = 0.70–0.93) and 1,146 subjects with MS and 1,309 healthy controls from Australia (rs2104286, *P* = 0.033; OR = 0.86; 95% c.i. = 0.75–0.99). In an extension analysis [15] using data from 12,360 subjects previously reported and new data from 11,019 unrelated MS subjects, 13,616 controls and 2,811 trio families (8,433 individuals) from across Europe, the association for MS risk at the two *IL2RA* variants became unequivocal (rs12722489, OR = 0.81 (95% c.i. 0.77–0.85), *P* = 2.24×10^−15^; relative risk, RR,  = 0.81 (95% c.i. 0.72–0.91), *P* = 5.47×10^−4^; rs2104286, OR = 0.80 (95% c.i. 0.77–0.84), *P* = 2.38×10^−23^; RR = 0.78 (95% c.i. 0.71–0.86)). Furthermore, this study demonstrated that rs2104286 is the primary association, and thus accounts for the association signal observed at rs12722489 [15].

For T1D susceptibility, two associations are known to exist at *IL2RA*. In a large-scale fine-mapping study of over 300 SNPs in the *IL2RA*-*RBM17* region in our T1D collection, we localized the association to T1D susceptibility to two groups of SNPs located in the 5′ region and intron 1 of *IL2RA*; any one or more SNPs from each group could potentially be the causal variant(s) [8]. The minor alleles at rs41295061 and rs11594656 were found to confer protection to T1D in a case-control DNA collection of 5,312 T1D subjects and 6,855 controls (rs41295061, OR,  = 0.65, rs11594656, OR = 0.87) and 2,612 families with T1D (rs41295061, RR = 0.70, rs11594656, RR = 0.89) [8].

The IL-2/IL-2RA(CD25) pathway plays an essential role in regulating immune responses [16]. IL-2 is central for both expansion and apoptosis of T cells, while high concentrations of soluble IL-2RA (sIL-2RA) are found in sera from healthy subjects and are increased in subjects with autoimmune disease, inflammation and infection [17]–[22]. Interestingly, we have previously shown that T1D-associated variants correlate with reduced levels of sIL-2RA [8].

Our knowledge of the IL-2R pathway and its central role in regulating immune responses prompted us to examine whether disease susceptibility at *IL2RA* to T1D and MS is due to shared or distinct genetic variants. First, we demonstrate extensive allelic heterogeneity between T1D and MS, including an allelic variant that is associated with susceptibility to one autoimmune disease but protection to the other. By extending previous genotype/phenotype correlations at *IL2RA*, we provide insight into both common and distinct functional mechanisms. Second, we extend our findings on the correlation between sIL-2RA levels and *IL2RA* genotype [8]. Using regression analyses, we show that sIL-2RA levels are determined by independent groups of SNPs, similar to what we show for disease susceptibility. Taken together, we demonstrate heterogeneity in the production of sIL-2RA in association with the genetic heterogeneity reported here. The approach described in this work will be instrumental for future investigations of complex causal mechanisms involved in human disease.

## Results/Discussion

**Figure 1:**
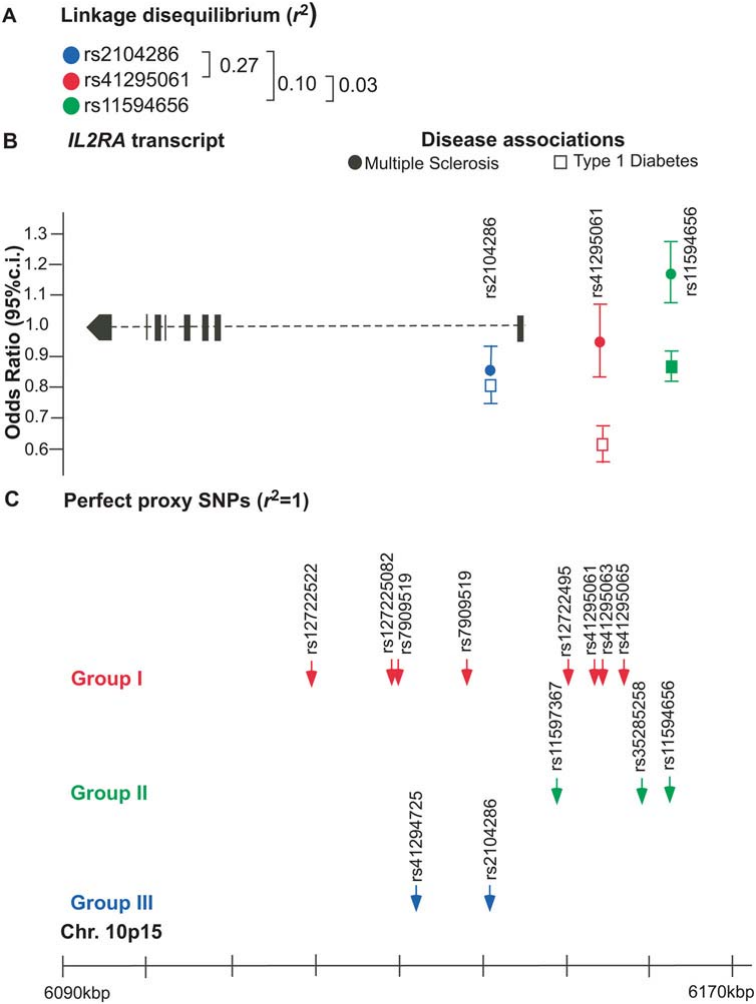
Association of *IL2RA* SNPs with multiple sclerosis and type 1 diabetes. (A) Linkage disequilibrium (*r* ^2^ values) between the SNPs in this study. *r* ^2^ values are based on 6,317 control subjects from Great Britain. (B) Disease associations (Odds Ratios of minor allele) with MS and T1D are shown for the three SNPs in this study. (C) The SNPs that are perfect proxies (*r* ^2^ = 1) for the SNPs studied are shown. These perfect proxy SNPs are based on the analysis of 32 CEPH individuals. MS, multiple sclerosis. T1D, type 1 diabetes.

The most associated *IL2RA* SNP for MS susceptibility is rs2104286 located in intron 1 of *IL2RA* [6],[15],[23],[24]. In the MS case-control and family collections we have analyzed, rs2104286 has an OR of 0.85 (95% c.i. 0.79–0.92, *P* = 6.27×10^−7^) (Table 1, Figure 1, Tables S1, S2, and S3). For T1D susceptibility, Lowe *et al.* [8] reported independent associations with two groups of indistinguishable SNPs, marked by rs41295061 (‘Group I’) and rs11594656 (‘Group II’) located in the 5′ region of the *IL2RA* gene. Here, we test these two SNPs for MS susceptibility. Single locus tests show no evidence of association between MS susceptibility and Group I (rs41295061; *P* = 0.10, Table 1). We note that assuming an effect size of rs41295061 as observed for T1D susceptibility (OR in the order of 0.6), the power to detect this effect is 97% in the parent/child trios and 100% in the MS case-control collection, given a significance level of 0.05 (Table S4, S5).

**Table 1:** Single locus analysis of the two MS-associated SNPs and the two T1D-associated SNPs in a DNA collection of up to 1,303 MS parent-child trios with MS from the USA, 2,440 MS cases from the USA, 6,425 T1D cases from GB and 9,407 healthy controls from the USA and GB.

	MS family and case-control collection					T1D case-control collection			
	Study population	T∶NT (% T) or n cases/n controls	MAF	RR or OR (95% c.i.)	P*	N cases/n controls	MAF	OR (95% c.i.)	*P*
rs41295061 C>A (marking Group I)	1,256 USA trios	204∶214 (48.8)	0.0921	0.95 (0.78–1.15)	0.6250	6,425/6,862	0.10	0.62 (0.56–0.68)	6.43×10^−25^
Minor	GB and USA case-control	2,382/9,141	0.1019	0.93 0.82–1.06)	0.2619				
	Combined				0.10				
rs11594656 T>A (marking Group II)	1,282 USA trios	539/505 (51.63)	0.2680	1.07 (0.95–1.21)	0.2930	6,425/6,862	0.25	0.87 (0.82–0.92)	3.37×10^−06^
	GB and USA case-control	2,205/8,891	0.2448	1.17 (1.07–1.27)	4.68×10^−4^				
	Combined				7.67×10^−4^				
rs2104286 A>G (marking Group III)	1,267 USA trios	400∶488 (45.05)	0.2500	0.84 (0.72–0.93)	3.15×10^−3^	6,425/6,862	0.28	0.80 (0.76–0.85)	1.27×10^−13^
	GB and USA case-control	2,420/9,407	0.2703	0.85 (0.79–0.92)	3.58×10^−5^				
	Combined				6.27×10^−7^				

Note that for rs2104286 and rs41295061 in the MS collection, we included previously published genotyping data (USA trios, US MS cases, US controls, GB cases; [6]). For rs41295061 and rs11594656 in the T1D collection, we included previously published genotyping data (GB cases and controls; [8]. We assumed a model of multiplicative effects when it was not significantly different from the full genotype model (*P*>0.05). For rs2104286, we used the full genotype model in the USA case-control collection, as it was significantly different from the multiplicative model (*P* = 6.57×10^−3^). Combined *P* values for the USA and GB case-control were stratified by population (note that for population stratification, a 2-df test was used for rs2104286 as there was a significant difference between 1-df and 2-df tests, *P* = 3.85×10^−3^). T, transmitted; NT, not transmitted; MAF, minor allele frequency in unaffected parents or control subjects; RR, relative risk of minor allele; OR, odds ratio of minor allele; 95% c.i. = 95% confidence interval.

Furthermore, Group II is associated with MS (rs11594656; *P* = 7.67×10^−4^ (Table 1, Figure 1). Surprisingly, at rs11594656, the minor allele A is associated with protection from T1D (OR = 0.87), but susceptibility to MS (OR = 1.17, Table 1) in the MS case-control collection. The lack of association in the parent/child trio collection may be due to low statistical power, which is only 31% for a variant with OR = 1.1 for this sample size and *P*<0.05 (Table S6).

The lack of MS association to Group I SNPs and the opposing effects associated with Group II SNPs indicates the presence of allelic heterogeneity between T1D and MS at Group I and Group II SNPs. In addition, we note that the MS-association observed at rs11594656 presents an independent MS-association from rs2104286 (Table S7). Taken together, rs2104286 marks an independent association from Group II SNPs (marked by rs11594656); we term this association ‘Group III’. Table S8 shows all *IL2RA* region SNPs in LD with rs2104286.

In order to explore the association of Group III SNPs to T1D susceptibility, we performed forward logistic regression analysis of the Group I, II and III SNPs in 6,425 T1D cases and 6,862 controls with complete genotyping data. The results are consistent with our previous study [8]: Group I has the strongest association with T1D (rs41295061, *P* = 6.43×10^−25^; Table 1). The first selected SNP in the regression analysis is rs41295061 and the second SNP to be added to the model including rs41295061 is rs11594656 (*P* = 2.07×10^−10^, Table S9). Interestingly, Group III also shows association with T1D (rs2104686, *P* = 1.27×10^−13^, Table 1). When we add rs2104286 to the model that includes both rs41295061 and rs11594656, this SNP adds to the model (*P* = 1.30×10^−5^; Table S10). These data indicate that rs2104286 (marking Group III) is independently associated with T1D. At rs2104286, it is the minor allele G of rs2104286 that confers protection from both MS and T1D (Table 1, Figure 1). We note here that the major allele at all T1D-associated loci discovered so far at *IL2RA* encodes the susceptibility allele.

Defining the heterogeneous genetic basis at *IL2RA* is critical for the success of functional studies aiming to connect the risk alleles with immunophenotypes and autoimmune mechanisms controlled by this locus. In a collection of T1D plasma samples, Lowe *et al.* [8] reported a correlation between the T1D *IL2RA* susceptibility alleles and decreased levels of sIL-2RA. This raised the possibility of a link between T1D susceptibility and the levels of this biomarker of peripheral inflammation [17]. Here, we investigate the correlation of sIL-2RA and the newly identified Group III SNPs, marked by rs2104286, which associates with both MS and T1D. In a replication study of up to 69 healthy control samples and 285 MS case samples we first confirm the previously observed correlation between rs11594656 with sIL-2RA levels; however, the low minor allele frequency of rs41295061 results in statistical power that was too low to detect the association with sIL-2RA in these sample collections (Table 2; Tables S11, S12). Most interestingly, however, an additional correlation between genotype and sIL-2RA level is observed at rs2104286 in our healthy control, MS and T1D collections, where the minor allele associates with decreased sIL-2RA levels. Given that the minor allele at rs2104286 associates with protection from both MS and T1D, this finding is unexpected because decreased sIL-2RA levels correlate with T1D susceptibility alleles at rs41295061 and rs11594656. This led us to investigate whether the three SNPs were marking independent associations with sIL-2RA levels, similarly to what we have observed for disease susceptibility at *IL2RA*. Indeed, using regression analyses in the T1D case collection, we show that the associations between sIL-2RA levels and rs11594656, rs41295061 and rs2104286 (Table 2) are independent from each other (Tables S14, S15).

**Table 2:** sIL-2RA concentrations in the sera of healthy controls and MS cases and plasma samples of T1D subjects.

SNP	Healthy controls			MS cases			T1D cases		
	N	Mean levels [ng/ml] (95% c.i.)	*P*	N	Mean levels [ng/ml] (95% c.i.)	*P*	N	Mean levels [ng/ml] (95% c.i.)	*P* *
**rs41295061**									
C/C	61	2.029 (1.843–2.214)		254	2.341 (2.248–2.433)		697	2.630 (2.555–2.705)	
C/A	7	1.946 (1.505–2.387)	0.98	28	2.351 (1.985–2.716)	0.68	263	2.938 (2.806–3.066)	2.7×10^−8^
A/A	1	2.328		3	3.656 (2.096–5.215)		18	1.886 (1.316–2.456)	
**rs11594656**									
T/T	40	1.770 (1.624–1.915)		163	2.222 (2.122–2.321)		821	2.486 (2.410–2.562)	
T/A	26	2.430 (2.088–2.772)	1.3×10^−3^	78	2.392 (2.223–2.562)	5.2×10^−4^	283	3.062 (2.923–3.203)	3.8×10^−19^
A/A	3	1.910 (1.039–2.780)		21	2.972 (2.515–3.428)		153	3.039 (2.839–3.238)	
**rs2104286**									
A/A	44	2.205 (1.971–2.439)		163	2.399 (2.289–2.510)		619	2.811 (2.713–2.909)	
A/G	22	1.805 (1.579–2.033)	4.6×10^−3^	86	2.017 (2.029–2.302)	6.0×10^−2^	462	2.574 (2.476–2.673)	1.0×10^−6^
G/G	5	1.463 (0.669–2.257)		26	1.654 (1.084–2.224)		86	2.281 (2.030–2.532)	

Analyses of log_10_-transformed sIL-2RA concentrations of healthy control, MS and T1D datasets. The analysis of sIL-2RA levels for rs41295061 and rs11594656 in up to 1,257 T1D cases presents a subset of a previously published data set [8]. Analyses were performed using a 2-degree of freedom test. We note that the healthy control and MS case collection present random population samples, but that individuals from the T1D case collection were chosen based on their genotype at both rs41295061 and rs11594656 to achieve representation of all genotypes for both SNPs [8]. This selection allowed the study of the correlation between sIL-2RA levels and the relatively rare minor allele at rs41295061, currently the most strongly associated T1D SNP (MAF = 0.09). N, number of samples.

*: T1D analyses were adjusted for covariates associated with log_10_-transformed sIL-2RA concentrations (Table S13).

Our combined genetic analyses of *IL2RA* variants in MS and T1D result in the discovery of a third, novel group of associated SNPs with T1D (Group III) and identifies a remarkable degree of allelic heterogeneity at this autoimmune susceptibility locus. This demonstrates the presence of (1) a T1D allele not associated with MS (rs41295061 marking Group I), (2) an allele conferring susceptibility to T1D but protection from MS (rs11594656 marking Group II) and (3) an allele shared between T1D and MS (rs2104286 marking Group III). The discovery of allelic heterogeneity between MS and T1D at *IL2RA* may only be a small window into the complexities that the *IL2RA* region harbors: GWA studies for both RA and SLE have also observed associations at *IL2RA* (Figure S1, Table S16); the overlap of associations among these and other diseases should be the focus of future studies. While any of the tested SNPs may be in LD with the true causal variant, the allelic heterogeneity we observe between MS and T1D provides strong evidence for the necessity of performing fine-mapping studies in each disease individually that associates with *IL2RA*. Another example of such allelic heterogeneity has been observed at the shared autoimmunity locus *PTPN22* encoding a lymphotyrosine phosphatase. While T1D, RA and CD all show disease associations that map to the same R620W variant (rs2476601) [25]–[27], it is the 620W variant that associates with risk to T1D and RA, but protection from CD [28]. We note that R620W has not shown association with MS susceptibility in the populations analyzed thus far [29],[30], but studies employing larger sample sizes will need to further address this variant in MS.

Our analysis of how disease susceptibility correlates with sIL-2RA levels suggest discordance between sIL-2RA level and disease susceptibility and calls for studies addressing causality of sIL-2RA in autoimmune disease. It is plausible that the three independent genetic associations marked by Group I to III SNPs present independent biological pathways that contribute to disease susceptibility. These pathways may involve transcriptional regulation of *IL2RA*, levels of surface expression of IL-2RA, in addition to serum sIL-2RA levels. In light of multiple, independent associations present at *IL2RA*, the genotype/phenotype correlations observed here and previously [8] may require extension to haplotype/phenotype correlations in sample sizes an order of magnitude greater than are currently available. Nevertheless, these data represent a comparative study between MS/T1D susceptibility and production of sIL-2RA and show that multiple variants contribute independently not only to disease susceptibility but also to an individual's sIL-2RA level.

## Materials and Methods

### Subjects

All case and control subjects were of self-reported white ethnicity and were enrolled under study protocols approved by the Institutional Review Board of each institution that contributed.

MS and T1D cases: Trio families and MS cases were collected as described in our recent investigation of patients with MS [31]. Subjects with MS all meet McDonald criteria for MS. T1D subjects were recruited as part of the Juvenile Diabetes Research Foundation/Wellcome Trust Diabetes and Inflammation Laboratory's British case collection (Genetic Resource Investigating Diabetes) [8], which is a joint project between the University of Cambridge Department of Pediatrics and the Department of Medical Genetics at the Cambridge Institute for Medical Research. Most cases were <16 years of age at the time of collection. All were under age 17 years at diagnosis, resided in Great Britain, and were of European descent (self-reported).

Healthy Control Subjects: Healthy adult control subjects were recruited through the Brigham and Women's Hospital and the University of California at San Francisco, as previously described [6]. They consisted of unrelated individuals who were self-reported as being of non-Hispanic white origin and having no history of chronic inflammatory disease. In addition, we included data from 1,679 control individuals collected throughout the USA as part of a GWAS of bipolar disorders sponsored by the NIMH (http://zork.wustl.edu/nimh). The GB control subjects were obtained from two collections, with 5,239 obtained from the British 1958 Birth Cohort, all born during one week in 1958 (National Child Development Study) and the remaining 1,445 controls selected from the UK Blood Services (UKBS) control collection [6]. All GB control subjects were of white ethnicity.

### Genotyping

SNPs were genotyped using the iPLEX Sequenom MassARRAY platform, TaqMan (Applied Biosystems), or MIP technology (Affymetrix) in accordance with the manufacturer's instructions. We analyzed only SNPs with high quality data (>95% genotype call rate, Hardy-Weinberg equilibrium in controls or unaffected parent *P*-value>0.001). MS collections were genotyped at the Broad Institute: rs41295061 and rs11594656 were genotyped using iPLEX Sequenom MassARRAY platform. The previously published data for the MS cases and healthy controls from the USA as well as the MS cases from GB were obtained from MIP technology [6]. GB healthy controls and GB T1D case were genotyped for rs2104286 using TaqMan genotyping at the Diabetes and Inflammation Laboratory. The previously published T1D data for rs41295061 and rs11594656 were obtained from TaqMan and MIP technology [8].

### sIL-2RA Measurements

ELISA measurement of sIL-2RA was performed according to the manufacturer's recommendations (BD Biosciences). Serum samples were diluted 1∶20 using PBS supplemented with 10% FBS. Microtiter plates were read using a Biorad Benchmark microplate reader. T1D plasma samples, healthy control and MS subject serum samples were stored at −80°C prior to analyses. A log_10_ transformation of total sIL-2RA concentration was used to provide a Normally distributed outcome. For T1D plasma samples, the analysis was adjusted for independently associated covariates, namely, age, duration of T1D and plasma storage duration. The healthy control subject population consisted of 60.3% females, 29.7% males, with an average age of 43 (range = 20–68) and an average sample storage duration of 2.1 years (range = 1.27–3.15). The MS subject population consisted of 74.2% females, 25.8% males, with an average age of 43 (range = 18–73) and an average sample storage duration of 2.4 (range = 1.1–3.3).

### Statistical Analysis

All statistical analyses were performed in either the Stata or R statistical systems. Single locus tests, logistic regression analyses, 2-d.f. locus-based tests were performed as described in [8]. Briefly, logistic regression analyses for the GB case-control collection were adjusted for 12 broad geographical regions within GB to minimize any confounding due to variation in allele frequencies across the country [32]. A multiplicative allelic effects model was assumed as it was not significantly different from the full genotype model for any of the SNPs (except for rs2104286 in the USA case-control collection, for which a full model was chosen as it was significantly different from the multiplicative model; *P* = 6.57×10^−3^). SNPs were modeled as a numerical indicator variable coded 0, 1 or 2, representing the number of occurrences of the minor allele. In the forward logistic regression analysis, we start by assessing the evidence against the most significant SNP being alone sufficient to model the association [33]. No specific mode of inheritance for the most associated SNP (A>a) or any additional SNP with significant independent effects of disease susceptibility was assumed, so genotype risks of A/A and A/a were modeled relative to the a/a genotype. Combined *P* values for the USA and GB case-control were stratified by population. Measures LD, D' *r* ^2^ were calculated using the Haploview package [34]. Power calculations were performed using the method described in [35].

## Supporting Information

Figure S1 Comparison of *IL2RA* variants genotyped in T1D, MS, RA and SLE. Minor allele associations with disease are shown. Odds ratios and 95% confidence intervals of association results are shown from the current study and previously published studies [8],[12],[14].
(0.11 MB DOC)

Table S1 Single-locus test *P* values for rs2104286, rs11594656 and rs41295061 in 2,115 MS cases and 6,902 healthy controls with complete genotype information (analysis stratified by population). MAF, minor allele frequency. OR, odds ratio.
(0.03 MB DOC)

Table S2 Single-locus test *P* values for rs2104286, rs11594656 and rs41295061 in 1,183 MS cases and 582 healthy controls from the USA with complete genotype information. MAF, minor allele frequency. OR, odds ratio.
(0.03 MB DOC)

Table S3 Single-locus test *P* values rs2104286, rs11594656 and rs41295061 in 932 MS cases and 6,320 healthy controls from GB with complete genotype information. MAF, minor allele frequency. OR, odds ratio.
(0.03 MB DOC)

Table S4 Power calculations to detect variants with odds ratios ranging from 1.1 to 1.4 and a minor allele frequency (MAF) of 0.10 using 1,250 parent/child trios. MAF, minor allele frequency. OR, odds ratio.
(0.03 MB DOC)

Table S5 Power calculations to detect the effect of variants with odds ratios ranging from 1.1 to 1.3 and a minor allele frequency (MAF) of 0.10 using 2,400 MS cases and 9,100 healthy controls. MAF, minor allele frequency. OR, odds ratio.
(0.03 MB DOC)

Table S6 Power calculations to detect the effect of variants with odds ratios (OR) ranging from 1.1 to 1.4 and a minor allele frequency (MAF) of 0.25 using 1,250 parent/child trios.
(0.03 MB DOC)

Table S7 Regression analysis (a) adding rs11594656 and rs41295061 to rs2104286 and reverse regression analysis (b) adding rs2104286 to rs11594656 and rs41295061 for 2,115 MS cases and 6,902 controls with complete genotype information (analysis stratified by population). ^1^ Results for a model assuming multiplicative effects and ^2^ for a model assuming genotype effects (full model) are shown. OR, odds ratio; *P*_diff_ = *P* value for tests between multiplicative and full models.
(0.05 MB DOC)

Table S8 LD between rs2104286 and all common variants located between 5.904,506 bp and 6.403,954 bp on chromosome 10. Positions are given using NCBI build 36. rs and in-house (DIL) SNP numbers are shown. LD measures are calculated based on 32 CEPH individuals; genotyping data are obtained from HapMap (www.hapmap.org) and [8].
(0.12 MB DOC)

Table S9 Regression analysis adding rs2104286 and rs11594656 to rs41295061 and regression analysis adding rs41295061 to rs2104286 and rs11594656 in 6,425 T1D cases and 6,862 controls. ^1^ Results for a model assuming multiplicative effects and ^2^ for a model assuming genotype effects (full model) are shown. OR, odds ratio; *P*_diff_ = *P* value for tests between multiplicative and full models.
(0.05 MB DOC)

Table S10 Regression analysis (a) adding rs2104286 to rs41295061 and rs11594656 and reverse regression analysis (b) adding rs41295061 and rs11584656 to rs2104286 in 6,425 T1D cases and 6,862 controls. ^1^ Results for a model assuming multiplicative effects and ^2^ for a model assuming genotype effects (full model) are shown. OR, odds ratio; *P*_diff_ = *P* value for tests between multiplicative and full models.
(0.06 MB DOC)

Table S11 Power calculations to detect the effect of variants with a minor allele frequency of 0.10 using 70 subjects. Power calculations were performed using the method described in [35].
(0.04 MB DOC)

Table S12 Power calculations to detect the effect of variants with a minor allele frequency of 0.10 using 280 subjects. Power calculations were performed using the method described in [35].
(0.04 MB DOC)

Table S13 Covariates associated with sIL-2RA concentrations in T1D analysis. We adjusted for the covariates year of birth, duration of disease and duration of storage of the plasma sample prior to processing, as these were all independently associated with log_10_-transformed sIL-2RA concentrations. Covariates were selected using forward and then reverse regression. The following covariates: gender, broad geographical region and the age and month when the plasma sample was collected all had *P*-values>0.05 when added to the selected covariates. Year of birth, duration of disease and the duration of storage of the plasma sample prior to processing were independently associated (*P*>0.05) and together account for 10.3% of the total variation with log_10_-transformed sIL-2RA concentration with the direction and magnitude shown in the table below. *%CV is the variation accountable by each covariate.
(0.04 MB DOC)

Table S14 Regression analysis adding rs2104286 and rs41295061 to rs11594656 and the reverse regression analysis adding rs11594656 to rs2104286 and rs41295061 in complete data for 1,167 T1D cases using log_10_-transformed sIL-2RA concentrations. 1 Results for a model assuming multiplicative effects and 2 for a model assuming genotype effects (full model) are shown. *P*_diff_ = *P* value for tests between multiplicative and full models.
(0.06 MB DOC)

Table S15 Regression analysis (a) adding rs2104286 to rs11594656 and rs41295061 and reverse regression analysis (b) adding rs11594656 to rs2104286 and rs41295061 and adding rs41295061 to rs2104286 and rs11594656 in complete data for 1,167 T1D cases using log_10_-transformed sIL-2RA concentrations.
(0.07 MB DOC)

Table S16 *r* ^2^ values for the four SNPs associated with T1D, MS or RA and the four SNPs studied in a GWA study for SLE susceptibility loci [8],[12],[14]. *r* ^2^ values are based on a maximum of 32 CEPH individuals. Allele frequencies are shown in the top diagonal line.
(0.05 MB DOC)
