# Supplementary material for: IL2RA Genetic Heterogeneity in Multiple Sclerosis and Type 1 Diabetes Susceptibility and Soluble Interleukin-2 Receptor Production
Source: PLoS Genet. 2009 Jan 2;5(1):e1000322. doi: 10.1371/journal.pgen.1000322 (PMC2602853; doi:10.1371/journal.pgen.1000322)
Supplement: Figure S1 — Comparison of IL2RA variants genotyped in T1D, MS, RA and SLE. Minor allele associations with disease are shown. Odds ratios and 95% confidence intervals of association results are shown from the current study and previously published studies [8],[12],[14]. (0.11 MB DOC) [file pgen.1000322.s001.doc]

**Figure S1:** Comparison of *IL2RA* variants genotyped in T1D, MS, RA and SLE. Minor allele associations with disease are shown. Odds ratios and 95% confidence intervals of association results are shown from the current study and previously published studies [8,12,14].
