# Supplementary material for: IL2RA Genetic Heterogeneity in Multiple Sclerosis and Type 1 Diabetes Susceptibility and Soluble Interleukin-2 Receptor Production
Source: PLoS Genet. 2009 Jan 2;5(1):e1000322. doi: 10.1371/journal.pgen.1000322 (PMC2602853; doi:10.1371/journal.pgen.1000322)
Supplement: Table S1 — Single-locus test P values for rs2104286, rs11594656 and rs41295061 in 2,115 MS cases and 6,902 healthy controls with complete genotype information (analysis stratified by population). MAF, minor allele frequency. OR, odds ratio. (0.03 MB DOC) [file pgen.1000322.s002.doc]

**Table S1:** Single-locus test *P* values for rs2104286, rs11594656 and rs41295061 in 2,115 MS cases and 6,902 healthy controls with complete genotype information (analysis stratified by population). MAF, minor allele frequency. OR, odds ratio.

| **Locus** | **MAF controls** | **OR**  **(95% c.i.)** | ***P*** |
| --- | --- | --- | --- |
| rs2104286 | 0.275 | 0.84 (0.77-0.93) | 3.0 x 10^-4^ |
| rs11594656 | 0.246 | 1.18 (1.08-1.29) | 4.1 x 10^-4^ |
| rs41295061 | 0.100 | 0.92 (0.80-1.06) | 0.24 |
