# Supplementary material for: IL2RA Genetic Heterogeneity in Multiple Sclerosis and Type 1 Diabetes Susceptibility and Soluble Interleukin-2 Receptor Production
Source: PLoS Genet. 2009 Jan 2;5(1):e1000322. doi: 10.1371/journal.pgen.1000322 (PMC2602853; doi:10.1371/journal.pgen.1000322)
Supplement: Table S2 — Single-locus test P values for rs2104286, rs11594656 and rs41295061 in 1,183 MS cases and 582 healthy controls from the USA with complete genotype information. MAF, minor allele frequency. OR, odds ratio. (0.03 MB DOC) [file pgen.1000322.s003.doc]

**Table S2:** Single-locus test *P* values for rs2104286, rs11594656 and rs41295061 in 1,183 MS cases and 582 healthy controls from the USA with complete genotype information. MAF, minor allele frequency. OR, odds ratio.

| **Locus** | **MAF controls** | **OR**  **(95% c.i.)** | ***P*** |
| --- | --- | --- | --- |
| rs2104286 | 0.263 | 0.78 (0.67-0.92) | 3.0 x 10^-4^ |
| rs11594656 | 0.250 | 1.18 (1.24-1.45) | 4.1 x 10^-4^ |
| rs41295061 | 0.092 | 0.81 (0.63-1.03) | 9.1 x 10^-2^ |
