# Supplementary material for: IL2RA Genetic Heterogeneity in Multiple Sclerosis and Type 1 Diabetes Susceptibility and Soluble Interleukin-2 Receptor Production
Source: PLoS Genet. 2009 Jan 2;5(1):e1000322. doi: 10.1371/journal.pgen.1000322 (PMC2602853; doi:10.1371/journal.pgen.1000322)
Supplement: Table S3 — Single-locus test P values rs2104286, rs11594656 and rs41295061 in 932 MS cases and 6,320 healthy controls from GB with complete genotype information. MAF, minor allele frequency. OR, odds ratio. (0.03 MB DOC) [file pgen.1000322.s004.doc]

**Table S3:** Single-locus test *P* values rs2104286, rs11594656 and rs41295061 in 932 MS cases and 6,320 healthy controls from GB with complete genotype information. MAF, minor allele frequency. OR, odds ratio.

| **Locus** | **MAF controls** | **OR**  **(95% c.i.)** | ***P*** |
| --- | --- | --- | --- |
| rs2104286 | 0.276 | 0.88 (0.78-0.98) | 2.0 x 10^-2^ |
| rs11594656 | 0.246 | 1.15 (1.03-1.28) | 1.6 x 10^-2^ |
| rs41295061 | 0.100 | 0.98 (0.83-1.15) | 0.76 |
