# Supplementary material for: IL2RA Genetic Heterogeneity in Multiple Sclerosis and Type 1 Diabetes Susceptibility and Soluble Interleukin-2 Receptor Production
Source: PLoS Genet. 2009 Jan 2;5(1):e1000322. doi: 10.1371/journal.pgen.1000322 (PMC2602853; doi:10.1371/journal.pgen.1000322)
Supplement: Table S4 — Power calculations to detect variants with odds ratios ranging from 1.1 to 1.4 and a minor allele frequency (MAF) of 0.10 using 1,250 parent/child trios. MAF, minor allele frequency. OR, odds ratio. (0.03 MB DOC) [file pgen.1000322.s005.doc]

**Table S4:** Power calculations to detect variants with odds ratios ranging from 1.1 to 1.4 and a minor allele frequency (MAF) of 0.10 using 1,250 parent/child trios. MAF, minor allele frequency. OR, odds ratio.

| **MAF = 0.10** | **Significance level** | | |
| --- | --- | --- | --- |
| OR | 0.05 | 0.01 | 0.001 |
| 1.1 | 18% | 6% | 1% |
| 1.2 | 52% | 28% | 10% |
| 1.3 | 83% | 64% | 36% |
| 1.4 | 97% | 89% | 69% |
