# Supplementary material for: IL2RA Genetic Heterogeneity in Multiple Sclerosis and Type 1 Diabetes Susceptibility and Soluble Interleukin-2 Receptor Production
Source: PLoS Genet. 2009 Jan 2;5(1):e1000322. doi: 10.1371/journal.pgen.1000322 (PMC2602853; doi:10.1371/journal.pgen.1000322)
Supplement: Table S5 — Power calculations to detect the effect of variants with odds ratios ranging from 1.1 to 1.3 and a minor allele frequency (MAF) of 0.10 using 2,400 MS cases and 9,100 healthy controls. MAF, minor allele frequency. OR, odds ratio. (0.03 MB DOC) [file pgen.1000322.s006.doc]

**Table S5:** Power calculations to detect the effect of variants with odds ratios ranging from 1.1 to 1.3 and a minor allele frequency (MAF) of 0.10 using 2,400 MS cases and 9,100 healthy controls. MAF, minor allele frequency. OR, odds ratio.

| **MAF = 0.10** | **Significance level** | | |
| --- | --- | --- | --- |
| **OR** | **0.05** | **0.01** | **0.001** |
| 1.1 | 67% | 43% | 18% |
| 1.2 | 100% | 98% | 91% |
| 1.3 | 100% | 100% | 100% |
