# Supplementary material for: IL2RA Genetic Heterogeneity in Multiple Sclerosis and Type 1 Diabetes Susceptibility and Soluble Interleukin-2 Receptor Production
Source: PLoS Genet. 2009 Jan 2;5(1):e1000322. doi: 10.1371/journal.pgen.1000322 (PMC2602853; doi:10.1371/journal.pgen.1000322)
Supplement: Table S7 — Regression analysis (a) adding rs11594656 and rs41295061 to rs2104286 and reverse regression analysis (b) adding rs2104286 to rs11594656 and rs41295061 for 2,115 MS cases and 6,902 controls with complete genotype information (analysis stratified by population). 1 Results for a model assuming multiplicative effects and 2 for a model assuming genotype effects (full model) are shown. OR, odds ratio; P diff = P value for tests between multiplicative and full models. (0.05 MB DOC) [file pgen.1000322.s008.doc]

**Table S7:** Regression analysis (a) adding rs11594656 and rs41295061 to rs2104286 and reverse regression analysis (b) adding rs2104286 to rs11594656 and rs41295061 for 2,115 MS cases and 6,902 controls with complete genotype information (analysis stratified by population).

^1^ Results for a model assuming multiplicative effects and ^2^ for a model assuming genotype effects (full model) are shown. OR, odds ratio; *P*_diff_ = *P* value for tests between multiplicative and full models.

| **Locus** |  | **Add locus to rs2104286** | |  | **Add rs2104286 to locus** | | | |
| --- | --- | --- | --- | --- | --- | --- | --- | --- |
|  |  | ***P*** | **OR (95% c.i.)** | ***P*_diff_** | ***P*** | **rs2104286** | **OR (95% c.i.)** | ***P*_diff_** |
| rs11594656 | A^1^ | 9.3 x 10^-3^ | 1.13 (1.03-1.25) | 0.76 | 3.9 x 10^-4^ | G^1^ | 0.82 (0.74-0.92) | 1.08x10^-2^ |
|  | T/A^2^ | 3.26x10^-2^ | 1.15 (1.01-1.30) |  | 1.09x10^-3^ | A/G^2^ | 0.79 (0.70-0.90) |  |
|  | A/A^2^ |  | 1.26 (1.00-1.59) |  |  | G/G^2^ | 0.93 (0.73-1.18) |  |
|  |  |  |  |  |  |  |  |  |
| rs41295061 | A^1^ | 0.36 | 1.08 (0.92-1.27) | 0.09 | 7.4 x 10^-3^ | G^1^ | 1.08 (0.92-1.28) | 4.33x10^-3^ |
|  | C/A^2^ | 0.166 | 1.14 (0.96-1.31) |  | 3.22 x 10^-5^ | A/G^2^ | 0.73 (0.64-0.84) |  |
|  | A/A^2^ |  | 0.70 (0.35-1.43) |  |  | G/G^2^ | 0.84 (0.65-1.08) |  |
