# Supplementary material for: IL2RA Genetic Heterogeneity in Multiple Sclerosis and Type 1 Diabetes Susceptibility and Soluble Interleukin-2 Receptor Production
Source: PLoS Genet. 2009 Jan 2;5(1):e1000322. doi: 10.1371/journal.pgen.1000322 (PMC2602853; doi:10.1371/journal.pgen.1000322)
Supplement: Table S8 — LD between rs2104286 and all common variants located between 5.904,506 bp and 6.403,954 bp on chromosome 10. Positions are given using NCBI build 36. rs and in-house (DIL) SNP numbers are shown. LD measures are calculated based on 32 CEPH individuals; genotyping data are obtained from HapMap (www.hapmap.org) and [8]. (0.12 MB DOC) [file pgen.1000322.s009.doc]

**Table S8:** LD between rs2104286 and all common variants located between 5.904,506 bp and 6.403,954 bp on chromosome 10. Positions are given using NCBI build 36. rs and in-house (DIL) SNP numbers are shown. LD measures are calculated based on 32 CEPH individuals; genotyping data are obtained from HapMap ([www.hapmap.org](http://www.hapmap.org/)) and [1].

| **rs#** | **DIL#** | **Position (bp)** | **Alleles** | **D' (rs2104286)** | ***r*^2^ (rs2104286)** |
| --- | --- | --- | --- | --- | --- |
| rs41294725 | DIL8126 | 6129785 | [-/ATTT] | 1 | 1 |
| rs41294713 | DIL9588 | 6120360 | [A/G] | 1 | 0.686 |
| rs12722489 | DIL8092 | 6142018 | [A/G] | 1 | 0.66 |
| rs791590 | DIL8124 | 6130328 | [A/T] | 1 | 0.66 |
| rs12722515 | DIL9589 | 6121236 | [G/T] | 1 | 0.66 |
| rs2246031 | DIL8115 | 6132216 | [A/G] | 1 | 0.628 |
| rs41295117 | DIL10041 | 6165328 | [C/T] | 0.848 | 0.627 |
| rs4747837 | DIL4593 | 6098741 | [C/G] | 0.845 | 0.549 |
| n/a | DIL4598 | 6098500 | [-/G] | 0.845 | 0.549 |
| rs41294697 | DIL8158 | 6109351 | [-/TTG] | 0.845 | 0.549 |
| rs7900385 | DIL8183 | 6102754 | [C/T] | 0.845 | 0.549 |
| rs11591302 | DIL9308 | 6091747 | [A/G] | 0.845 | 0.549 |
| rs4625363 | DIL9558 | 6112510 | [C/T] | 0.845 | 0.549 |
| rs12722527 | DIL9573 | 6117334 | [A/G] | 0.845 | 0.549 |
| rs12722523 | DIL9582 | 6118396 | [C/T] | 0.845 | 0.549 |
| rs12722551 | DIL9640 | 6111385 | [A/G] | 0.838 | 0.537 |
| rs12722553 | DIL9642 | 6111290 | [G/T] | 0.838 | 0.537 |
| rs7093069 | DIL8176 | 6103325 | [A/G] | 0.819 | 0.497 |
| rs12722574 | DIL8164 | 6106468 | [C/T] | 0.723 | 0.462 |
| rs7900744 | DIL8167 | 6105617 | [C/T] | 0.723 | 0.462 |
| rs12722581 | DIL8170 | 6104622 | [-/T] | 0.723 | 0.462 |
| rs11597237 | DIL8150 | 6119023 | [A/G] | 0.723 | 0.462 |
| rs12722585 | DIL8180 | 6102935 | [-/CTCA] | 0.723 | 0.462 |
| rs12722588 | n/a | 6100439 | [A/G] | 0.723 | 0.462 |
| rs12722587 | DIL8190 | 6100636 | [A/G] | 0.721 | 0.458 |
| rs41294671 | DIL8441 | 6089803 | [A/G] | 0.797 | 0.458 |
| rs17147986 | DIL9353 | 6078484 | [A/C] | 1 | 0.455 |
| rs41295055 | DIL10022 | 6151628 | [C/T] | 0.718 | 0.454 |
| rs41294709 | DIL9572 | 6116415 | [A/-] | 0.712 | 0.446 |
| rs11256416 | DIL9568 | 6115365 | [A/G] | 0.665 | 0.442 |
| n/a | DIL13218 | 6167647 | [A/G] | 1 | 0.432 |
| rs12722529 | DIL9571 | 6116517 | [T/-] | 1 | 0.428 |
| rs41295077 | DIL10005 | 6158268 | [A/G] | 0.626 | 0.392 |
| rs41295079 | DIL10055 | 6159160 | [C/T] | 0.626 | 0.392 |
| rs41295105 | DIL10862 | 6162680 | [G/T] | 0.626 | 0.392 |
| n/a | DIL13878 | 6161291 | [A/G] | 0.626 | 0.392 |
| rs2182410 | DIL10861 | 6162675 | [C/T] | 1 | 0.371 |
| rs7078614 | DIL9567 | 6115837 | [A/C] | 1 | 0.356 |
| rs3118475 | DIL10007 | 6147271 | [C/T] | 0.592 | 0.35 |
| rs7078273 | DIL13895 | 6168553 | [G/T] | 1 | 0.345 |
| rs41295075 | DIL10004 | 6158252 | [C/T] | 0.782 | 0.34 |
| rs41295103 | DIL10849 | 6162201 | [A/G] | 0.782 | 0.34 |
| rs12722517 | DIL9591 | 6121046 | [A/G] | 0.613 | 0.335 |
| rs809356 | DIL8121 | 6131154 | [A/G] | 1 | 0.317 |
| rs62626317 | DIL13870 | 6150931 | [A/G] | 0.594 | 0.295 |
| rs764851 | DIL9279 | 6087950 | [A/T] | 0.593 | 0.295 |
| rs791588 | DIL8130 | 6129348 | [A/C] | 1 | 0.275 |
| rs41295071 | DIL10001 | 6158117 | [C/T] | 1 | 0.263 |
| rs41295113 | DIL10035 | 6163335 | [A/G] | 1 | 0.263 |
| rs41295081 | DIL10056 | 6159240 | [C/T] | 1 | 0.263 |
| rs41295099 | DIL10907 | 6161729 | [TAA/-] | 1 | 0.263 |
| rs41295061 | DIL10023 | 6154666 | [A/C] | 1 | 0.263 |
| rs41295049 | DIL10871 | 6149682 | [A/G] | 1 | 0.263 |
| rs7909519 | DIL8125 | 6129847 | [A/C] | 1 | 0.263 |
| rs12722522 | DIL9584 | 6118559 | [C/T] | 1 | 0.263 |
| rs12722495 | DIL9620 | 6137289 | [C/T] | 1 | 0.263 |
| rs12722508 | DIL8131 | 6128749 | [A/T] | 1 | 0.262 |
| rs12722558 | DIL8152 | 6110282 | [A/T] | 0.744 | 0.252 |
| rs12722563 | DIL8157 | 6109567 | [C/T] | 0.744 | 0.252 |
| rs791593 | DIL9596 | 6123298 | [C/T] | 0.669 | 0.248 |
| rs11596136 | n/a | 6081879 | [C/T] | 0.729 | 0.236 |
| rs7078535 | DIL10844 | 6160430 | [C/T] | 1 | 0.229 |
| rs706778 | DIL8104 | 6138955 | [A/G] | 1 | 0.229 |
| rs11256601 | DIL10015 | 6159631 | [C/T] | 1 | 0.226 |
| rs34410111 | DIL10830 | 6191268 | [A/G] | 0.668 | 0.224 |
| rs791587 | DIL8132 | 6128705 | [C/T] | 1 | 0.223 |
| rs41295065 | DIL10026 | 6156981 | [A/G] | 1 | 0.22 |
| rs41295063 | DIL10029 | 6155210 | [A/G] | 1 | 0.22 |
| rs7072793 | DIL10879 | 6146272 | [C/T] | 1 | 0.2 |
| rs7073236 | DIL10880 | 6146558 | [C/T] | 1 | 0.2 |
| rs7096384 | DIL10883 | 6146644 | [C/T] | 1 | 0.2 |
| rs10795791 | DIL4575 | 6148346 | [C/T] | 1 | 0.2 |
