# Supplementary material for: IL2RA Genetic Heterogeneity in Multiple Sclerosis and Type 1 Diabetes Susceptibility and Soluble Interleukin-2 Receptor Production
Source: PLoS Genet. 2009 Jan 2;5(1):e1000322. doi: 10.1371/journal.pgen.1000322 (PMC2602853; doi:10.1371/journal.pgen.1000322)
Supplement: Table S9 — Regression analysis adding rs2104286 and rs11594656 to rs41295061 and regression analysis adding rs41295061 to rs2104286 and rs11594656 in 6,425 T1D cases and 6,862 controls. 1 Results for a model assuming multiplicative effects and 2 for a model assuming genotype effects (full model) are shown. OR, odds ratio; P diff = P value for tests between multiplicative and full models. (0.05 MB DOC) [file pgen.1000322.s010.doc]

**Table S9:** Regression analysis adding rs2104286 and rs11594656 to rs41295061 and regression analysis adding rs41295061 to rs2104286 and rs11594656 in 6,425 T1D cases and 6,862 controls.

^1^ Results for a model assuming multiplicative effects and ^2^ for a model assuming genotype effects (full model) are shown. OR, odds ratio; *P*_diff_ = *P* value for tests between multiplicative and full models.

| **Locus** |  | **Add locus to rs41295061** | |  | **Add rs41295061 to locus** | | | |
| --- | --- | --- | --- | --- | --- | --- | --- | --- |
|  |  | ***P*** | **OR (95% c.i.)** | ***P*_diff_** | ***P*** | **rs41295061** | **OR (95% c.i.)** | ***P*_diff_** |
| rs2104286 | G^1^ | 7.34 x 10^-3^ | 0.91 (0.86-0.98) | 0.27 | 1.69 x 10^-14^ | A^1^ | 0.66 (0.60-0.74) | 0.75 |
|  | A/G^2^ | 1.53 x 10^-2^ | 0.89 (0.82-0.97) |  | 1.58 x 10^-13^ | C/A^2^ | 0.67 (0.60-0.75) |  |
|  | G/G^2^ |  | 0.88 (0.75-1.04) |  |  | A/A^2^ | 0.41 (0.25-0.66) |  |
|  |  |  |  |  |  |  |  |  |
| rs11594656 | A^1^ | 2.07 x 10^-10^ | 0.82 (0.78-0.87) | 0.92 | 4.89 x 10^-29^ | A^1^ | 0.59 (0.54-0.65) | 0.97 |
|  | T/A^2^ | 1.68 x 10^-9^ | 0.83 (0.77-0.89) |  | 6.90 x 10^-28^ | C/A^2^ | 0.83 (0.77-0.89) |  |
|  | A/A^2^ |  | 0.67 (0.57-0.79) |  |  | A/A^2^ | 0.67 (0.57-0.79) |  |
