# Supplementary material for: IL2RA Genetic Heterogeneity in Multiple Sclerosis and Type 1 Diabetes Susceptibility and Soluble Interleukin-2 Receptor Production
Source: PLoS Genet. 2009 Jan 2;5(1):e1000322. doi: 10.1371/journal.pgen.1000322 (PMC2602853; doi:10.1371/journal.pgen.1000322)
Supplement: Table S10 — Regression analysis (a) adding rs2104286 to rs41295061 and rs11594656 and reverse regression analysis (b) adding rs41295061 and rs11584656 to rs2104286 in 6,425 T1D cases and 6,862 controls. 1 Results for a model assuming multiplicative effects and 2 for a model assuming genotype effects (full model) are shown. OR, odds ratio; P diff = P value for tests between multiplicative and full models. (0.06 MB DOC) [file pgen.1000322.s011.doc]

**Table S10:** Regression analysis (a) adding rs2104286 to rs41295061 and rs11594656 and reverse regression analysis (b) adding rs41295061 and rs11584656 to rs2104286 in 6,425 T1D cases and 6,862 controls.

^1^ Results for a model assuming multiplicative effects and ^2^ for a model assuming genotype effects (full model) are shown. OR, odds ratio; *P*_diff_ = *P* value for tests between multiplicative and full models.

| **(a)** | **Add locus to rs41295061 and rs11594656** | | | |
| --- | --- | --- | --- | --- |
| **Locus** |  | ***P*** | **OR (95% c.i.)** | ***P*_diff_** |
| rs2104286 | G^1^ | 1.30x10^-5^ | 0.86 (0.80-0.92) | 0.28 |
|  | A/G^2^ | 4.22x10^-6^ | 0.84 (0.77-0.91) |  |
|  | G/G^2^ |  | 0.78 (0.66-0.92) |  |
|  |  |  |  |  |
|  |  |  |  |  |
| **(b)** | **Add locus to rs41295061 and rs2104286** | | | |
| **Locus** |  | ***P*** | **OR (95% c.i.)** | ***P*_diff_** |
| rs11594656 | A^1^ | 5.14x10^-13^ | 0.80 (0.75-0.85) | 0.84 |
|  | T/A^2^ | 4.64 x10^-12^ | 0.80 (0.74-0.86) |  |
|  | A/A^2^ |  | 0.63 (0.53-0.74) |  |
|  |  |  |  |  |
|  | **Add locus to rs2104286 and rs11594656** | | | |
| **Locus** |  | ***P*** | **OR (95% c.i.)** | ***P*_diff_** |
| rs41295061 | A^1^ | 5.68 x10^-15^ | 0.66 (0.59-0.73) | 0.76 |
|  | C/A^2^ | 5.39 x10^-14^ | 0.66 (0.59-0.74) |  |
|  | A/A^2^ |  | 0.40 (0.25-0.65) |  |
