# Supplementary material for: IL2RA Genetic Heterogeneity in Multiple Sclerosis and Type 1 Diabetes Susceptibility and Soluble Interleukin-2 Receptor Production
Source: PLoS Genet. 2009 Jan 2;5(1):e1000322. doi: 10.1371/journal.pgen.1000322 (PMC2602853; doi:10.1371/journal.pgen.1000322)
Supplement: Table S11 — Power calculations to detect the effect of variants with a minor allele frequency of 0.10 using 70 subjects. Power calculations were performed using the method described in [35]. (0.04 MB DOC) [file pgen.1000322.s012.doc]

**Table S11:** Power calculations to detect the effect of variants with a minor allele frequency of 0.10 using 70 subjects. Power calculations were performed using the method described in [35].

| **70 samples** | **Significance level** | | |
| --- | --- | --- | --- |
| **Variance explained (%)** | **0.05** | **0.01** | **1x10^-3^** |
| 0.5 | 9% | 2% | 0.3% |
| 1 | 13% | 4% | 0.6% |
| 2 | 21% | 8% | 1.7% |
| 3 | 30% | 12% | 3% |
| 4 | 38% | 18% | 5% |
| 5 | 45% | 23% | 7% |
| 6 | 52% | 29% | 10% |
| 7 | 59% | 35% | 13% |
| 8 | 65% | 41% | 17% |
| 9 | 70% | 46% | 21% |
| 10 | 74% | 52% | 25% |
