# Supplementary material for: IL2RA Genetic Heterogeneity in Multiple Sclerosis and Type 1 Diabetes Susceptibility and Soluble Interleukin-2 Receptor Production
Source: PLoS Genet. 2009 Jan 2;5(1):e1000322. doi: 10.1371/journal.pgen.1000322 (PMC2602853; doi:10.1371/journal.pgen.1000322)
Supplement: Table S13 — Covariates associated with sIL-2RA concentrations in T1D analysis. We adjusted for the covariates year of birth, duration of disease and duration of storage of the plasma sample prior to processing, as these were all independently associated with log10-transformed sIL-2RA concentrations. Covariates were selected using forward and then reverse regression. The following covariates: gender, broad geographical region and the age and month when the plasma sample was collected all had P-values>0.05 when added to the selected covariates. Year of birth, duration of disease and the duration of storage of the plasma sample prior to processing were independently associated (P>0.05) and together account for 10.3% of the total variation with log10-transformed sIL-2RA concentration with the direction and magnitude shown in the table below. *%CV is the variation accountable by each covariate. (0.04 MB DOC) [file pgen.1000322.s014.doc]

**Table S13:** Covariates associated with sIL-2RA concentrations in T1D analysis. We adjusted for the covariates year of birth, duration of disease and duration of storage of the plasma sample prior to processing, as these were all independently associated with log_10_-transformed sIL-2RA concentrations.  Covariates were selected using forward and then reverse regression. The following covariates: gender, broad geographical region and the age and month when the plasma sample was collected all had *P-*values > 0.05 when added to the selected covariates. Year of birth, duration of disease and the duration of storage of the plasma sample prior to processing were independently associated (*P* > 0.05) and together account for 10.3% of the total variation with log_10_-transformed sIL-2RA concentration with the direction and magnitude shown in the table below.

| **Covariate** | **Unit** | P | **%CV*** | **Log_10_ sIL-2RA levels:** |
| --- | --- | --- | --- | --- |
| Year of birth | years | 1.84 x10^-19^ | 6.00 | increase with year of birth (decrease with age) |
| Duration of Disease | months | 5.96 x10^-8^ | 2.12 | increase with duration of disease |
| Duration of storage of  plasma prior to processing | months | 2.50 x10^-10^ | 2.90 | decrease with storage time |

*%CV is the variation accountable by each covariate.
