# Supplementary material for: IL2RA Genetic Heterogeneity in Multiple Sclerosis and Type 1 Diabetes Susceptibility and Soluble Interleukin-2 Receptor Production
Source: PLoS Genet. 2009 Jan 2;5(1):e1000322. doi: 10.1371/journal.pgen.1000322 (PMC2602853; doi:10.1371/journal.pgen.1000322)
Supplement: Table S14 — Regression analysis adding rs2104286 and rs41295061 to rs11594656 and the reverse regression analysis adding rs11594656 to rs2104286 and rs41295061 in complete data for 1,167 T1D cases using log10-transformed sIL-2RA concentrations. 1 Results for a model assuming multiplicative effects and 2 for a model assuming genotype effects (full model) are shown. P diff = P value for tests between multiplicative and full models. (0.06 MB DOC) [file pgen.1000322.s015.doc]

**Table S14:** Regression analysis adding rs2104286 and rs41295061 to rs11594656 and the reverse regression analysis adding rs11594656 to rs2104286 and rs41295061 in complete data for 1,167 T1D cases using log_10_-transformed sIL-2RA concentrations.

^1^ Results for a model assuming multiplicative effects and ^2^ for a model assuming genotype effects (full model) are shown. *P*_diff_ = *P* value for tests between multiplicative and full models.

| **Locus** |  | **Add locus to rs11594656** | |  | **Add rs11594656 to locus** | | | |
| --- | --- | --- | --- | --- | --- | --- | --- | --- |
|  |  | ***P*** | **Coefficient (95% c.i.)** | ***P*_diff_** | ***P*** | **rs11594656** | **Coefficient (95% c.i.)** | ***P*_diff_** |
| rs41295061 | A^1^ | 0.044 | 0.02 (0.001-0.05) | 4.67 x 10^-5^ | 1.27 x 10^-14^ | A^1^ | 0.06 (0.04-0.07) | 1.81 x 10^-6^ |
|  | C/A^2^ | 4.00 x 10^-5^ | 0.15 (0.06-0.24) |  | 1.40 x 10^-18^ | T/A^2^ | 0.03 (0.01-0.07) |  |
|  | A/A^2^ |  | 0.10 (0.02-0.19) |  |  | A/A^2^ | 0.08 (0.05-0.11) |  |
|  |  |  |  |  |  |  |  |  |
| rs2104286 | G^1^ | 8.61 x 10^-5^ | 0.04 (0.02-0.50) | 0.867 | 4.62 x 10^-10^ | A^1^ | 0.05 (0.03-0.06) | 2.77 x 10^-10^ |
|  | A/G^2^ | 4.42 x 10^-4^ | 0.03 (0.01-0.05) |  | 8.29 x 10^-18^ | A/T^2^ | 0.11(0.09-0.14) |  |
|  | G/G^2^ |  | 0.07(0.03-0.11) |  |  | A/A^2^ | 0.05 (0.01-0.08) |  |
