# Supplementary material for: IL2RA Genetic Heterogeneity in Multiple Sclerosis and Type 1 Diabetes Susceptibility and Soluble Interleukin-2 Receptor Production
Source: PLoS Genet. 2009 Jan 2;5(1):e1000322. doi: 10.1371/journal.pgen.1000322 (PMC2602853; doi:10.1371/journal.pgen.1000322)
Supplement: Table S15 — Regression analysis (a) adding rs2104286 to rs11594656 and rs41295061 and reverse regression analysis (b) adding rs11594656 to rs2104286 and rs41295061 and adding rs41295061 to rs2104286 and rs11594656 in complete data for 1,167 T1D cases using log10-transformed sIL-2RA concentrations. (0.07 MB DOC) [file pgen.1000322.s016.doc]

**Table S15:**

Regression analysis (a) adding rs2104286 to rs11594656 and rs41295061 and reverse regression analysis (b) adding rs11594656 to rs2104286 and rs41295061 and adding rs41295061 to rs2104286 and rs11594656 in complete data for 1,167 T1D cases using log10-transformed sIL-2RA concentrations.

^1^ Results for a model assuming multiplicative effects and ^2^ for a model assuming genotype effects (full model) are shown. *P*_diff_ = *P* value for tests between multiplicative and full models.

(a)

| **Locus** |  | **Add locus to rs11594656 and rs41295061** | |  |
| --- | --- | --- | --- | --- |
|  |  | ***P*** | **Coefficient (95% c.i.)** | ***P*_diff_** |
| rs2104286 | G^1^ | 1.21 x 10^-9^ | 0.06 (0.04-0.08) | 0.265 |
|  | A/G^2^ | 5.09 x 10^-9^ | 0.70 (0.05-0.09) |  |
|  | G/G^2^ |  | 0.11(0.06-0.16) |  |
|  |  |  |  |  |
| **(b)** |  |  |  |  |
| **Locus** |  | **Add locus to rs2104286 and rs41295061** | |  |
|  |  | ***P*** | **Coefficient (95% c.i.)** | ***P*_diff_** |
| rs11594656 | A^1^ | 6.36 x 10^-9^ | 0.04 (0.03-0.06) | 7.48 x 10^-7^ |
|  | T/A^2^ | 2.29 x 10^-13^ | 0.04 (0.01-0.08) |  |
|  | A/A^2^ |  | 0.05 (0.02-0.08) |  |
|  |  |  |  |  |
| **Locus** |  | **Add locus to rs2104286 and rs11594656** | |  |
|  |  | ***P*** | **Coefficient (95% c.i.)** | ***P*_diff_** |
| rs41295061 | A^1^ | 4.42 x 10^-7^ | 0.07(0.04-0.10) | 2.89 x 10^-5^ |
|  | C/A^2^ | 4.42 x 10^-10^ | 0.12 (0.03-0.21) |  |
|  | A/A^2^ |  | 0.02 (0.07-0.12) |  |
