# Supplementary material for: IL2RA Genetic Heterogeneity in Multiple Sclerosis and Type 1 Diabetes Susceptibility and Soluble Interleukin-2 Receptor Production
Source: PLoS Genet. 2009 Jan 2;5(1):e1000322. doi: 10.1371/journal.pgen.1000322 (PMC2602853; doi:10.1371/journal.pgen.1000322)
Supplement: Table S16 — r 2 values for the four SNPs associated with T1D, MS or RA and the four SNPs studied in a GWA study for SLE susceptibility loci [8],[12],[14]. r 2 values are based on a maximum of 32 CEPH individuals. Allele frequencies are shown in the top diagonal line. (0.05 MB DOC) [file pgen.1000322.s017.doc]

**Table S16:** *r*^2^ values for the four SNPs associated with T1D, MS or RA and the four SNPs studied in a GWA study for SLE susceptibility loci [8,12,14]. *r*^2^ values are based on a maximum of 32 CEPH individuals. Allele frequencies are shown in the top diagonal line.

|  | **rs2104286** | **rs12722489** | **rs7072793** | **rs4147359** | **rs7090530** | **rs11594656** | **rs12251307** | **rs41295061** |
| --- | --- | --- | --- | --- | --- | --- | --- | --- |
| **rs2104286** | **0.83** |  |  |  |  |  |  |  |
| **rs12722489** | 0.66 | **0.89** |  |  |  |  |  |  |
| **rs7072793** | 0.2 | 0.13 | **0.52** |  |  |  |  |  |
| **rs4147359** | 0.12 | 0.08 | 0.6 | **0.61** |  |  |  |  |
| **rs7090530** | 0 | 0.07 | 0.48 | 0.31 | **0.64** |  |  |  |
| **rs11594656** | 0.04 | 0.02 | 0.32 | 0.18 | 0.62 | **0.79** |  |  |
| **rs12251307** | 0.07 | 0 | 0 | 0.05 | 0.03 | 0.02 | **0.92** |  |
| **rs41295061** | 0.26 | 0.01 | 0.05 | 0.03 | 0.1 | 0.01 | 0.38 | **0.95** |
